# Liposomal TriCurin, A Synergistic Combination of Curcumin, Epicatechin Gallate and Resveratrol, Repolarizes Tumor-Associated Microglia/Macrophages, and Eliminates Glioblastoma (GBM) and GBM Stem Cells

**DOI:** 10.3390/molecules23010201

**Published:** 2018-01-18

**Authors:** Sumit Mukherjee, Juliet N. E. Baidoo, Samay Sampat, Andrew Mancuso, Lovena David, Leah S. Cohen, Shuiqin Zhou, Probal Banerjee

**Affiliations:** 1 Ph.D. Program in Biochemistry, The Graduate Center, City University of New York, New York, NY 10016, USA; smukherjee@gradcenter.cuny.edu (S.M.); Juliet.Baidoo@csi.cuny.edu (J.N.E.B.); Andrew.Mancuso@csi.cuny.edu (A.M.); 2 Department of Chemistry, City University of New York, The College of Staten Island, Staten Island, NY 10314, USA; Leah.Cohen@csi.cuny.edu (L.S.C.); Shuiqin.Zhou@csi.cuny.edu (S.Z.); 3 College of Arts and Science, New York University, New York, NY 10003, USA; samay.sampat@gmail.com; 4 Center for Developmental Neuroscience, City University of New York, The College of Staten Island, Staten Island, NY 10314, USA; lovenagrace@yahoo.com

**Keywords:** curcumin, epicatechin gallate, resveratrol, TriCurin, liposomes, glioblastoma multiforme (GBM), GBM stem cells, tumor-associated microglia/macrophages (TAM), NK cells

## Abstract

Glioblastoma (GBM) is a deadly brain tumor with a current mean survival of 12–15 months. Despite being a potent anti-cancer agent, the turmeric ingredient curcumin (C) has limited anti-tumor efficacy in vivo due to its low bioavailability. We have reported earlier a strategy involving the use two other polyphenols, epicatechin gallate (E) from green tea and resveratrol (R) from red grapes at a unique, synergistic molar ratio with C (C:E:R: 4:1:12.5, termed TriCurin) to achieve superior potency against HPV+ tumors than C alone at C:E:R (μM): 32:8:100 (termed 32 μM+ TriCurin). We have now prepared liposomal TriCurin (TrLp) and demonstrated that TrLp boosts activated p53 in cultured GL261 mouse GBM cells to trigger apoptosis of GBM and GBM stem cells in vitro. TrLp administration into mice yielded a stable plasma concentration of 210 nM C for 60 min, which, though sub-lethal for cultured GL261 cells, was able to cause repolarization of M2-like tumor (GBM)-associated microglia/macrophages to the tumoricidal M1-like phenotype and intra-GBM recruitment of activated natural killer cells. The intratumor presence of such tumoricidal immune cells was associated with concomitant suppression of tumor-load, and apoptosis of GBM and GBM stem cells. Thus, TrLp is a potential onco-immunotherapeutic agent against GBM tumors.

## 1. Introduction

Glioblastoma Multiforme (GBM) is a pernicious form of primary brain tumor, which chiefly arises from malignancy of astrocytes and constitutes approximately 15% of adult primary brain tumor cases [1,2,3]. Th average survival of patients with established GBM is about 12–15 months from diagnosis despite the current standard of care, which includes surgical removal of the tumor bulk followed by radiation and chemotherapy to eliminate residual tumor cells. Subsequently the patient undergoes a 5-day chemotherapy regimen with a high-dose of temozolomide (TMZ) every 28 days [4,5,6,7]. Unfortunately, neurotoxicity resulting from mechanical traumas due to surgery, radiotherapy and chemotherapy can increase extracellular concentrations of glutamine, which nourishes the GBM cells and triggers tumor proliferation [8]. Additionally, radiation can cause tumor recurrence by triggering proliferation of brain tumor stem cells [9]. Furthermore, long-term TMZ treatment results in severe lymphopenia and immune suppression in the patients, which can promote tumor progression [10,11,12,13,14,15,16,17]. Survival of GBM patients without treatment is usually 3 months from diagnosis [18]. Thus there is an acute need for alternative, tumor-specific therapeutic agents, which are non-toxic to normal tissue.

Curcumin (C), a polyphenolic component of turmeric, is potent against a wide spectrum of tumors including GBM, both in vitro and in vivo [13,15,19,20,21,22,23,24,25,26,27,28,29]. Unfortunately, C’s in vivo anti-tumor potency is severely limited by its poor bioavailability, which is understood to be due to low its plasma levels, inadequate tissue distribution, rapid metabolism, hydrophobicity, and short half-life [30,31]. Over the years, our group has held the position that the anti-tumor activity of C can be significantly improved by enhancing its bioavailability through stabilization, targeting and modification using various innovative methods [14,20,21,22,26,27,32]. One such strategy, as reported in our previous studies, used “Combination Index Analysis” to demonstrate that combining C with two other food-derived natural polyphenols, epicatechin gallate (E) from green tea and resveratrol (R) from red grapes at a unique ratio, (C:E:R: 32:8:100), yields a synergistic formulation termed as 32 µM+ TriCurin with higher potency than C, E, or R against human and murine HPV+ cervical cancer cells in vitro, possibly through the induction of activated-p53 [26,27]. Our data also indicated that this novel combination is more stable than C and is non-toxic to healthy tissue [26,27]. Furthermore, we showed that TriCurin stabilizes and/or efficiently promotes intra-tumor uptake of C to cause elimination of HPV+ tumors [26,27]. Multiple reports have also established that liposomal encapsulation of C (CLp) enhances C’s solubility, stability, bioavailability and anti-tumor efficacy in vivo compared to free C [33,34,35,36]. Based on such evidence, we aimed to further enhance the stability and blood-brain-barrier permeability of TriCurin by preparing liposome-encapsulated TriCurin (TrLp) and then test its anti-tumor efficacy on GBM tumor cells in vitro and in vivo in comparison with CLp.

We demonstrate here that the TrLp-triggered mechanism of elimination of GL261 (mouse GBM) cells and GBM stem cells in vitro may involve activated-p53-mediated growth suppression and apoptosis. However, the in vivo (plasma) concentration of TrLp-associated-C was lower than its IC_50_ for cultured GL261 cells, yet it was able to cause a dramatic decrease in tumor-load via elimination of GBM and GBM stem cells and tumor remission in a pilot experiment in 50% of mice with established GBM [36]. As a possible mechanism of this anti-tumor activity of TrLp, we observed that even at this low plasma concentration of TrLp-associated-C, TrLp was capable of causing repolarization of the pro-tumor M2-like tumor-associated microglia/macrophages (TAM) to the tumoricidal M1-like phenotype and intra-tumor recruitment of activated Natural Killer Cells (NK cells) [12,14,15,16,17,37,38]. This was associated with an induction in activated caspase3 in the GBM cells and suppression of GBM stem cells in the tumors. Thus, TrLp is a potential onco-immunotherapeutic agent against established GBM tumors [2,3,4,11,12,26,27].

## 2. Results

### 2.1. Physical Characterization of CLp and TrLp Particles

**Figure 1:**
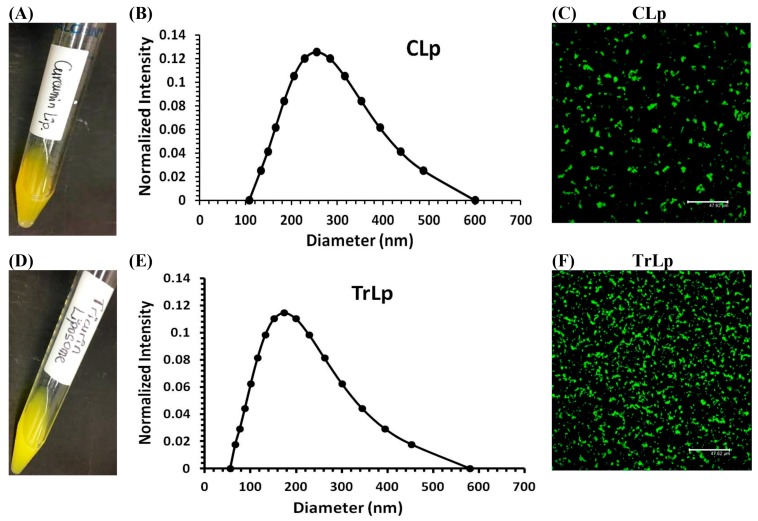
Physical Characterization of CLp and TrLp particles. (**A**,**D**) CLp (5 mM) and TrLp (1.28 mM+) exhibited a light-yellow, stable emulsion with no noticeable precipitation upon long-term standing; (**B**,**E**) Based on DLS analysis, the median hydrodynamic diameters were ~260 nm. (*n* = 3) for CLp and ~200 nm for TrLp; (**C**,**F**) Confocal laser scanning at 540 nm (C’s emission maximum) revealed spherical CLp and TrLp particles.

Both CLp (5 mM) and TrLp (1.28 mM+) appeared as light-yellow, uniform emulsions in PBS with no insoluble C or TriCurin (Figure 1A,D). The appearances of our emulsions (Figure 1A,D) indicated encapsulation of C and TriCurin, possibly through the formation of amphipathic liposomal particles [33,34,35,36]. Dynamic light scattering (DLS) analysis revealed a median hydrodynamic diameter of ~260 nm for CLp and ~200 nm for TrLp (Figure 1B,E). Liposomes in these diameter ranges are reportedly useful for medical applications such as drug delivery [39]. Confocal laser scanning at 540 nm (C’s emission) revealed the presence of C in both CLp and TrLp, (Figure 1C,F).

### 2.2. TrLp Is More Potent Than CLp in Eliminating GL261 Cells and Inhibiting Its Clonogenic Potential

In WST-1 assays, TriCurin displayed a 3.75-fold lower IC_50_ than C alone for the GL261 cells. Similar results were obtained when comparing TrLp and CLp, where TrLp exhibited a 3.2-fold lower IC_50_ than CLp (Table S1). Cells treated with free liposomes (control) showed no discernible cyto-toxicity (Mukherjee et al., unpublished data). After purging with nitrogen and storing at −20 °C for 14 days, TrLp and CLp faithfully replicated the IC_50_ values (Table S1), thus demonstrating the stability of the liposomes at −20 °C.

To compare the signaling potency of TrLp and CLp in cultured GL261 cells, we treated the cells for 6 h with vehicle or TrLp or CLp at the following concentrations: TrLp (10 µM+) and CLp (10 µM), which lies midway between the IC_50_ of TrLp (5 µM+) (i.e., 5 µM TrLp-associated C) and the IC_50_ of CLp (16 µM) (Table S1) [26]. The effects of TrLp (10 µM+) and CLp (10 µM) on the clone-generating potency of 96-h-treated and surviving GL261 cells relative to the vehicle-treated cells were assessed after 15 days [27]. CLp reduced the number of clonogenic (tumorigenic) colonies by 38%, whereas TrLp reduced the number of clonogenic colonies by 88% (Figure S1A–D). Thus, TrLp has a greater anti-tumor capacity than CLp, probably due to greater stabilization and/or increased intake of C from TriCurin into the tumor cells [26,27].

Our earlier studies on GL261 and HPV+ tumor cells identified NF-κB as a major target of C and TriCurin [22,26]. C is known to inhibit p65 NF-κB expression in tumor cells, causing attenuation of NF-κB -mediated suppression of p300-HAT. The subsequent increase in p300-HAT is expected to cause acetylation-mediated activation and stabilization of the tumor-suppressor protein p53 [40,41,42]. p53 upregulation and activation would elicit downstream activation of caspase3, thereby triggering apoptosis in the tumor cells [26]. To further delineate the mechanistic underpinnings of TrLp-mediated elimination of GL261 cells, we performed immunostaining and flow cytometry analysis of cultured GL261cells treated with CLp (10 µM), TrLp (10 µM+), and Vehicle (PBS) for 6 h [26].

### 2.3. TrLp Potently Upregulates Activated p53 in Cultured GL261 Cells

In flow cytometry analysis and comparison of antibody staining for a particular antigen, we have expressed our results as integrated fluorescence (IF), which is a product of the immunofluorescence emitted by each cell and the total number of cells expressing a specific antigen (IF = fluorescence from each cell × total number of cells in a specific stained population). 

**Figure 2:**
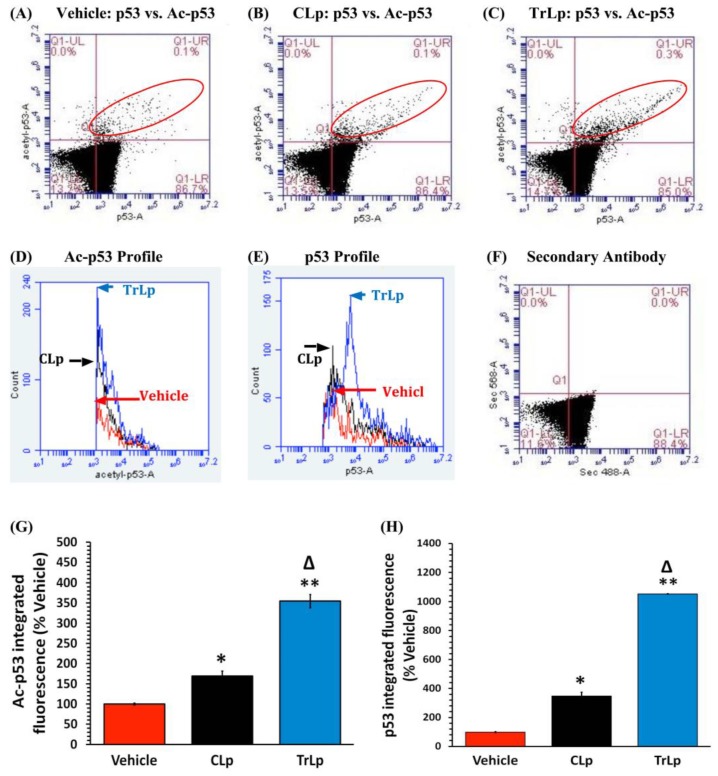
TrLp is significantly more potent than CLp in boosting activated p53 in cultured GL261 cells. Flow cytometry analysis of Acetyl-p53+ and p53+ events (double-stained cells in the upper right quadrant (UR) indicated by red ellipses) revealed that CLp treatment increased acetyl-p53 integrated fluorescence (IF) by 70% (* *p* = 0.03) and the p53 IF by 248% (* *p* = 0.01) with respect to the Vehicle-treated (**A**,**B**,**D**,**E**,**G**,**H**); In contrast, TrLp boosted acetyl-p53 IF by 255% (** *p* = 4.2 × 10^−3^) and p53 IF by 954% (** *p* = 1.6 × 10^−5^) in comparison with the Vehicle group (**A**,**C**–**E**,**G**,**H**). This elevation acetyl-p53 IF was 185% higher (Δ *p* = 0.012) and that for p53 was 406% higher (Δ *p* = 1.5 × 10^−3^) for TrLp group than for the CLp group (**B**–**E**,**G**,**H**); Data (mean ± S.E.M.) were obtained from Vehicle (*n* = 4), CLp (*n* = 4), and TrLp (*n* = 4). Integrated fluorescence (IF) = mean fluorescence per cell × total number of cells (events) in a segregated population; (**F**) 2° antibody-treated samples showed background staining.

Such analysis of cells double-stained with antibodies against acetyl p53 and p53 (upper right quadrant, within the red ellipse) revealed that CLp increased acetyl-p53 IF by 70% compared to the vehicle-treated GL261 cells, whereas TrLp caused a dramatic elevation in acetyl-p53 IF by 255% (Figure 2A–D,G). Additionally, CLp was able to boost the p53 level by 248% with respect to vehicle-treated. In contrast, TrLp caused a striking upregulation in p53 level by 954% (Figure 2A–C,E,H). Thus, in cultured GL261 cells, TrLp caused an increase in p53 activity through a combination of induction in expression as well as acetylation of p53.

### 2.4. TrLp-Evoked Induction in Activated p53 Is Associated with caspase3 Activation in CD68(+) GL261 Cells

**Figure 3:**
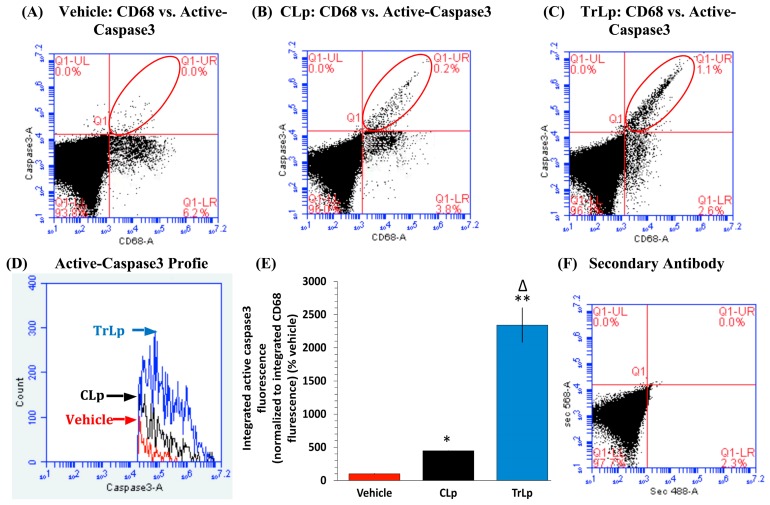
TrLp is significantly more potent than CLp in causing activation of caspase3 in CD68(+) GL261 cells. Flow cytometry analysis of treated-GL261 cells showed two discrete populations of GL261 cells: CD68+ and active-caspase3+ (double-stained cells) (apoptotic) enclosed within red ellipses in the UR quadrant and single-stained CD68(+) but active-caspase3(−) (non-apoptotic) appearing in the lower right (LR) quadrant. (**A**,**D**,**E**) The Vehicle-treated CD68+ GL261 cells harbored minimal active-caspase3+ (apoptotic) cells (UR) and many active-caspase3(−) cells (non-apoptotic) (LR). (**A**,**B**,**D**,**E**) Compared to the Vehicle-treated, the CLp-treated CD68(+) cells (apoptotic, UR, red ellipse) showed a 348% increase in active-caspase3 IF (* *p* = 7.7 × 10^−5^) (**D**). (**A**,**C**–**E**) This increase in active-capsase3 IF was 2243% in the TrLp-treated cells (UR, red ellipse) (** *p* = 0.01) (Δ *p* = 0.02, TrLp versus CLp) (**D**). Data (mean ± S.E.M.) obtained from Vehicle (*n* = 3), CLp (*n* = 3) and TrLp (*n* = 3). (**F**) 2° antibody-treated samples showed background staining.

Our prior studies have revealed that GL261 cells (and other GBM cell lines) express high levels of CD68, which was used as a marker for therapeutic targeting in vitro and in vivo [14,22]. Thus, we used flow cytometry to analyze the possibility of caspase3 activation in the CD68+ GL261 cells possibly as a consequence of TrLp and CLp-evoked increase in active p53 (Figure 2) [26]. As expected, CLp caused a 348% increase in active-caspase3 integrated fluorescence (IF) in the CD68+ GL261 cells relative to vehicle-treated cells (active-caspase3+, CD68+ cells in the UR quadrant within the red ellipse) (Figure 3A,B,D,E). In contrast, this increase in active-caspase3 IF was 2243% for TrLp (1895% more than CLp) (Figure 3A,C–E). It should be noted that CD68(+) but caspase3(−) non-apoptotic cells in the lower right quadrant decreased parallel to an increase in CD68(+), active-caspase3(+) (double-stained) cells in the UR quadrant.

### 2.5. TrLp-Evoked Suppression of CD133(+) and SOX2(+) GL261 Stem Cells in Culture

**Figure 4:**
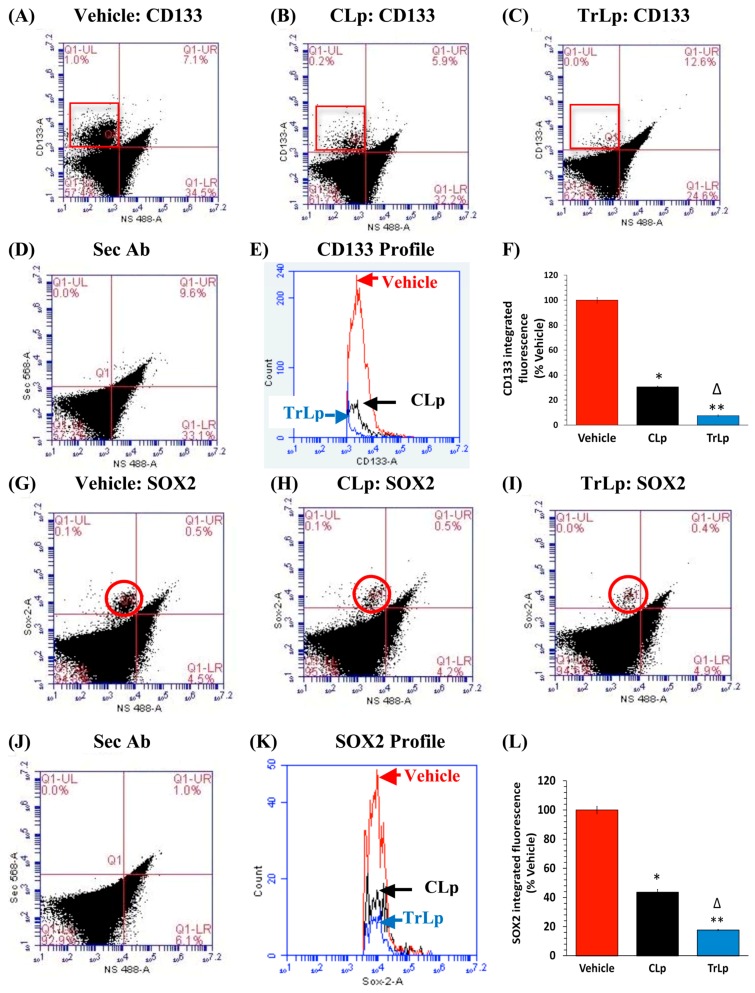
TrLp is significantly more potent than CLp in causing suppression of CD133(+) and SOX2(+) GL261 stem cells. Treated GL261 cells were stained with antibodies against CD133 and SOX2 and analyzed via flow cytometry. (**A**,**E**,**F**) The Vehicle-treated GL261 cells showed an abundance of CD133(+) GBM stem cells (UL quadrant, within the red rectangle). (**A**–**C**,**E**,**F**) Compared to the Vehicle-treated, the CLp-treated cells (UL quadrant, within the red rectangle) showed a 69% suppression of CD133 IF (* *p* = 1.2 × 10^−3^) and TrLp-treated cells (UL quadrant, within the red rectangle) showed a 92% suppression of CD133(+) IF (** *p* = 6.9 × 10^−4^) (**E**,**F**); (**F**) TrLp treatment yielded 23% greater suppression of CD133 IF than CLp (Δ *p* = 1.6 × 10^−3^); (**D**) 2° antibody staining showed background fluorescence; (**G**–**I**,**K**,**L**) Compared to the Vehicle-treated cells, the CLp-treated cells (UL quadrant, within the red circle) showed 56% suppression of SOX2 IF (* *p* = 3.2 × 10^−3^), and the TrLp-treated cells showed 82% suppression of SOX2 IF (** *p* = 1.0 × 10^−3^) (**K**,**L**); (**L**) This inhibition was 26% greater in the TrLp group than in the CLp group (Δ *p* = 5.6 × 10^−3^). Data (mean ± S.E.M.) were obtained from Vehicle (*n* = 3), CLp (*n* = 3) and TrLp (*n* = 3); (**J**) 2° antibody-treated samples showed background staining.

GL261 cells are known to express a small subset of rarely-dividing, chemo- and radio-resistant, CD133(+) and SOX2(+) GBM stem cells. GBM stem cells are responsible for tumor recurrence, and C is known to inhibit these GBM stem cells [9,17,23,24,43,44]. Interestingly, it has also been shown that reactivation of p53 (similar to our observation in Figure 2) in breast cancer cells causes a reduction in the number of tumor stem cells [45]. Thus in order to compare the effects of TrLp and CLp on GBM stem cells, we single-stained treated-GL261 cells with antibodies against the stem cell markers CD133 and SOX2 and performed flow cytometry analysis. CLp suppressed the CD133(+) IF by 69% (UL, red square) (Figure 4A,B,E,F), whereas TrLp reduced it by 92% (Figure 4A,C,E,F). Similarly, CLp caused a decrease in SOX2 integrated fluorescence by 56% (UL, red circle) (Figure 4G,H,K,L), whereas TrLp caused a dramatic 82% reduction in SOX2 IF (Figure 4G,I,K,L).

### 2.6. TrLp Causes Increased Internalization of TrLp-Associated Curcumin in SOX2(+) GL261 Stem Cells in Culture

Based on our prior report, TriCurin causes greater stabilization and/or cellular uptake of C compared to free C [26]. To explore its effect on GBM stem cell elimination, we performed immunocytochemistry to detect SOX2(+) GL261 stem cells from the three groups after treatment (Vehicle, CLp and TrLp) and looked for intra-cellular C (emission at 540 nm, green) in SOX2(+) cells (Figure S2). Cells of the Vehicle group displayed sparse populations of SOX2(+) GL261 stem cells. (Figure S2A,B). CLp treatment caused a 78% decrease in SOX2 fluorescence (Figure S2A,B), and a 296% increase in C’s fluorescence in the SOX2(+) cells (Figure S2A,C). TrLp-treatment induced a 94% suppression in SOX2 fluorescence (Figure S2A,B) while causing a 1947% increase in C’s fluorescence in the SOX2(+) cells (Figure S2A,C). Additionally, CLp and TrLp caused 114% and 878% increase in C accumulation respectively in the GL261 cells in general (Figure S2A,D). This may indicate that the higher anti-GBM efficacy of TrLp could be due to its ability to elicit relatively higher intracellular accumulation of C than caused by CLp.

### 2.7. Pharmacokinetic Assessment of Curcuminoids (C) in the Plasma of TrLp-Injected Mice by HPLC

Before translating the superior anti-tumor potency of TrLp obtained from in vitro studies (Table S1 and Figure S1) into in vivo settings with a pre-clinical GL261-implanted GBM mouse model [14], it was essential to assess the bioavailability and pharmacokinetics of C in the plasma of TrLp-treated tumor-naive mice (*n* = 3 per time point). Low stability and bioavailability of free C seriously impedes its antitumor efficacy [28,30,31]. Pharmacokinetic analysis plasma C using high performance liquid chromatography (HPLC) revealed that after 10 min from i.p. injection of 200 µL of 1.28 mM+ TrLp (containing 0.256 micromoles of C), the curcuminoid level in plasma reached its peak (0.61 µM) (Figure S3). Interestingly, the plasma level of C stabilized from 20 to 60 min, showing a steady concentration of ~0.2 µM even at 1 h from injection. Intriguingly, this level is ~11 fold higher than the previously reported value where oral gavage of 340 mg/kg of Curcumin Phytosome Meriva^®^ yielded a plasma concentration of ~0.019 µM (~7 ng/mL) in rat, 1 h from delivery [36]. These data and the results from our in vitro observation that TriCurin enhances the stability and/or uptake of C into tumor cells (Figure S2) [26] support the possibility that curcumin delivery in the form of TrLp increases its bioavailability and stability than in the form of CLp, and is thus a potentially superior anti-tumor formulation than CLp in vivo.

Though the observed plasma concentration of TrLP-derived C was higher than that reported earlier, it was still below the IC_50_ of TriCurin-associated C for the GL261 cells (5 μM). The intrabrain concentrations of TrLp-derived C was expected to be even lower than the plasma concentrations of C [28]. Although our earlier studies elucidated the likely mechanism of elimination of such cervical cancer cells in vitro [26,27], it was not clear how the low plasma concentration of TriCurin-derived C could eliminate cancer cells in vivo. It was also far from clear if such low TriCurin-derived C could eliminate established glioblastoma brain tumors. 

Since plasma concentration C from TrLp was lower than the IC_50_ for TriLp-derived C in vitro, we sought to study if TrLp causes stimulation of the innate immune cells to eliminate GBM and GBM stem cells in vivo [13,37,46,47,48]. To this end, we used the immunocompetent GL261-implanted GBM mice [14,49]. The GL261-implanted GBM mice with established tumors were randomly divided into three groups (*n* = 3 for each group) and intra-peritoneally (i.p.) treated with Vehicle (PBS) or TrLp (1.28 mM+) or CLp (5 mM) (see Methods) every 24 h for five days from day 12 after GL261-implantation on day 1 [14,22,32]. On day 17, the mice were sacrificed, their GBM-containing brains extricated, and each GBM brain was divided into two parts for immunohistochemistry (IHC) and flow cytometry as discussed in the Methods section.

### 2.8. In a Short-Term (5-Day) Treatment Regimen, TrLp and CLp Cause Repolarization of iNOS^low^ Arg1^high^ M2-Like Tumor-Associated Microglia/Macrophages (TAM) to iNOS^high^ Arg1^low^ M1-Like TAM and Intra-Tumor Recruitment of NKp46(+) (Activated) Natural Killer (NK) Cells

**Figure 5:**
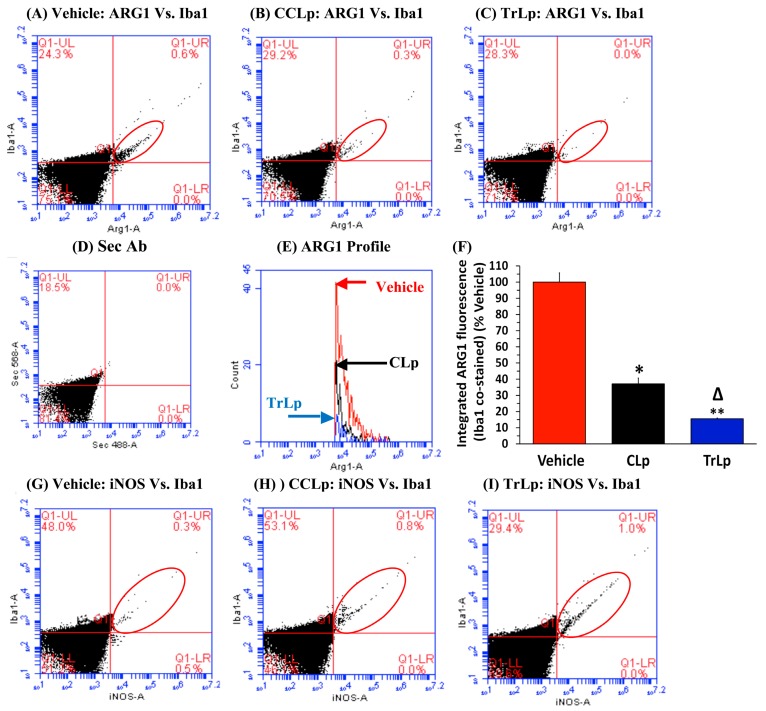

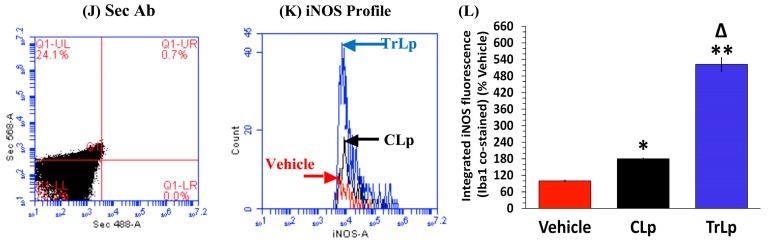
A 5-day treatment of GBM mice shows that TrLp is significantly more potent than CLp in causing repolarization of iNOS^low^, Arg1^high^ M2-like TAM to iNOS^high^, Arg1^low^ M1-like TAM. GL261 cells (10^5^) were implanted in the right brain of 3-month old male C57BL/6 mice on day 1 by stereotaxic injection and randomly divided into three groups: Vehicle, CLp (5 mM) and TrLp (1.28 mM+). The GBM mice from the three groups were treated (i.p. 200 µL Vehicle, CLp, or TrLp) from day 12 to day 16 (five times, every 24 h) and sacrificed on day 17. Dispersed GBM tumors from mice of the three groups were double-stained with antibodies against Iba1 and ARG1 or iNOS (double-stained events indicated by red ellipses in the UR quadrant) and analyzed via flow cytometry. (**A**,**E**,**F**) The Iba1(+) TAM in the Vehicle-treated, were ARG1^high^ (M2); (**A**–**C**,**E**,**F**) Compared to the Vehicle-treated, the CLp-treated, Iba1(+) TAM (UR, red ellipse) showed 63% less ARG1 IF (* *p* = 8 × 10^−4^) and the TrLp-treated TAM (UR, red ellipse) showed 84% less ARG1 IF (** *p* = 1.3 × 10^−4^) (**E**,**F**); (**F**) This suppression was 21% greater in the TrLp mice than in the CLp cohort (Δ *p* = 4.4 × 10^−3^); (**D**) 2° antibody-treated samples showed background staining; (**G**,**K**,**L**) The Iba1(+) TAM in the Vehicle-treated GBM mice were iNOS^low^ (M2 type); (**G**–**I**,**K**,**L**) Compared to the Vehicle-treated, the CLp-treated, Iba1(+) TAM (UR, red ellipse) showed 80% greater the iNOS IF (* *p* = 1.4 × 10^−4^) and the TrLp-treated TAM showed 422% higher iNOS IF (** *p* = 1.5 × 10^−4^) (**K**,**L**); (**L**) This increase iNOS was 342% greater in the TrLp-treated than in the CLp cohort (Δ *p* = 0.001); The graphs represent data (mean ± S.E.M.) obtained from Vehicle (*n* = 3 mice), CLp (*n* = 3 mice) and TrLp (*n* = 3 mice); (**J**) 2° antibody-treated samples showed background staining.

In our earlier report, we had demonstrated that the TAM display weak CD68 staining but strong Iba1 expression (CD68^low^ and Iba1(+)). In contrast, the tumor cells express high levels of CD68 but no Iba1 protein (CD68^high^ and Iba1(−)) [22]. To examine the likelihood of TrLp-evoked repolarization of pro-tumor ARG1^high^/iNOS^low^-M2-like TAM into anti-tumor ARG1^low^/iNOS^high^-M1-like Iba1(+) TAM, we performed flow cytometry analysis of immunostained cells dissociated from vehicle-, CLp(−) and TrLp-treated GBM tumors (Figure 5) [14].

The tumor-associated Iba1(+) cells (activated microglia/macrophages) (UR, red ellipse) in the vehicle-treated mice were ARG1^high^/iNOS^low^ (M2-like phenotype) (Figure 5A,E–G,K,L), whereas in the CLp-treated and TrLp-treated tumors, the Iba1(+) cells were ARG1^low^/iNOS^high^ (M1-like TAM phenotype) (Figure 5B,C,E,F,H,I,K,L). We observed a 63% decrease in ARG1 IF (Figure 5A,B,E,F) and an 80% increase in iNOS IF in the Iba1+ TAM of the CLp group (Figure 5G,H,K,L). In contrast the TAM from the TrLp group displayed an 84% decrease in ARG1 IF (Figure 5A,C,E,F) and a 422% increase in iNOS IF (Figure 5G,I,K,L).

**Figure 6:**
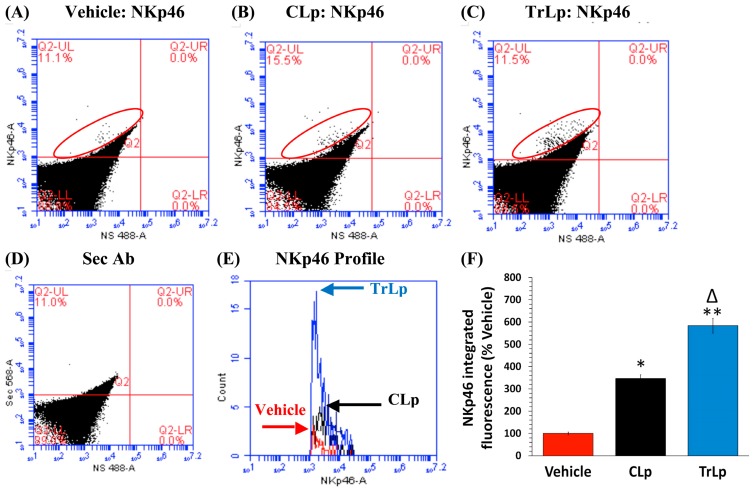
After a 5-day treatment of GBM mice, TrLp causes higher NK Cell recruitment than CLp. (**A**,**E**,**F**) GBM tumors from Vehicle-treated, GBM mice contained very small number of NKp46(+) activated NK cells; (**A**–**C**,**E**,**F**) Compared to the Vehicle-treated group, the CLp-treated samples (UL, red ellipse) showed a 247% increase in NKp46 IF (* *p* = 1.8 × 10^−4^) and the TrLp-treated samples displayed 484% higher NKp46 IF (** *p* = 3.7 × 10^−4^) (**E**,**F**); (**F**) Thus the TrLp group showed 237% higher augmentation NKp46 IF than the CLp mice (Δ *p* = 8.0 × 10^−3^). Data (mean ± S.E.M.) obtained from Vehicle (*n* = 3 mice), CLp (*n* = 3 mice) and TrLp (*n* = 3 mice); (**D**) 2° antibody-treated samples showed background staining.

These observations were further confirmed by IHC analysis of brain sections harboring GBM tumors from the three groups (Vehicle, CLp, and TrLp), collecting tissues parallel to the samples used for flow cytometry analyses shown in Figure 5 [14]. The Iba1(+) TAM in the Vehicle-treated GBM sections showed ARG1^high^/iNOS^low^ phenotype (Figure S4A–C). The CLp-treated mice exhibited a 67% decrease in ARG1 and 192% increase in iNOS Figure S4A–C). In contrast, the TAM in the TrLp group displayed an 82% decrease in ARG1 and a 393% increase in iNOS (Figure S4A–C). We next investigated the possibility of TrLp- and CLp-triggered, recruitment of activated NK cells into the tumor (Figure 6) [50,51].

Based on flow cytometry analysis of dissociated tumor cells (UL, red ellipse), CLp-treatment caused a 247% increase in NKp46 IF (Figure 6A,B,E,F) and TrLp-treated samples exhibited a 484% increase in NKp46 IF (Figure 6A,C,E,F). This demonstrates greater infiltration of activated NK cells into the GBM tumors in response to TrLp treatment.

These observed changes (in Figure 6) were verified using IHC analysis of sections parallel to those used in Figure S4. We observed the presence of only sparse NKp46+ activated-NK cells in the Vehicle-treated GBM sections (Figure S5A,B). The CLp-treated group showed a 264% increase in NKp46 staining (Figure S5A,B) and the TrLp-treated sections showed a 553% increase in NKp46 (Figure S5A,B).

### 2.9. In the 5-Day Treatment of GBM Mice, TrLp and CLp Cause Suppression of CD133(+) GBM Stem Cells and Apoptosis of GL261-Evoked CD68^high^ GBM Tumor Cells

**Figure 7:**
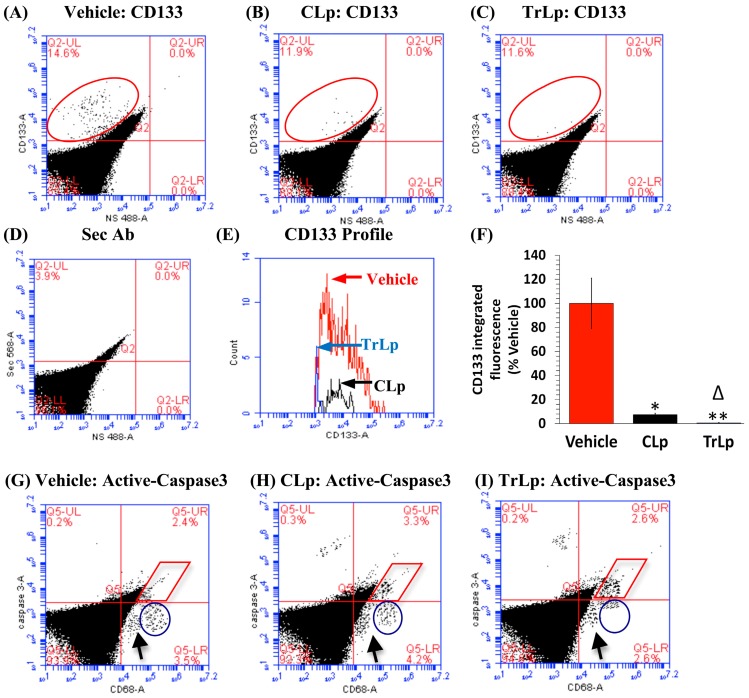

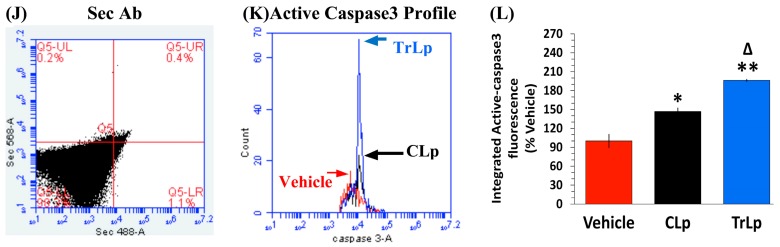
Short-term treatment of GBM mice with TrLp and CLp causes suppression of GBM stem cells and apoptosis of GBM cells. (**A**–**L**) Dispersed GBM tumor cells from five-day-treated mice of the three groups were stained with antibodies against CD133 and analyzed by flow cytometry. (**A**,**E**,**F**) The Vehicle-treated mice showed a number of CD133(+) GBM stem cells (UL, within the red ellipse); (**A**–**C**,**E**,**F**) Relative to Vehicle-treated, the CLp-treated mice showed a 92% suppression of CD133 IF (* *p* = 0.03) and the TrLp-treated samples displayed a 99% suppression of CD133 IF (** *p* = 5.0 × 10^−3^) (**E**,**F**); (**B**) 2° antibody-treated samples showed background staining; (**G**–**L**) Dispersed cells double-stained for CD68 and Active-Caspase3 showed two discrete populations: CD68^high^, Active-Caspase3(+) (apoptotic GBM cells, UR within the red rhombus) and CD68^high^, Active-Caspase3(−) (non-apoptotic GBM cells, LR within blue circles) [14,22]; (**G**,**K**,**L**) A few of the Vehicle-treated CD68^high^ GBM cells are Active-Caspase3(+) (within red rhombus), but a large number were Active-Caspase3(−) cells (non-apoptotic, LR within blue circle); (**G**,**H**,**K**,**L**) CLp treatment caused a 47% increase in Active-Caspase3 IF (**K**,**L**) of CD68^high^, Active-Caspase3(+) cells (apoptotic, UR within red rhombus) (* *p* = 0.04; versus Vehicle-treated), along with a decrease in the number of CD68^high^, Active-Caspase3(−) cells (LR, blue circle); (**G**,**I**,**K**,**L**) TrLp treatment caused a 96% increase in Active-Capsase3 IF (**K**,**L**) of CD68^high^ cells (** *p* = 0.04, TrLp versus Vehicle-treated) along with a further decrease in CD68^high^, Active-Caspase3(−) cells (LR, blue circle). In contrast, the levels of the CD68^low^ cells shown by black arrows (likely TAM) did not change appreciably; (**K**,**L**) This increase in Active-Caspase3 in the TrLp group was 49% greater than in the CLp-treated (Δ *p* = 0.02). Data (mean ± S.E.M.) obtained from Vehicle (*n* = 3 mice), CLp (*n* = 3 mice) and TrLp (*n* = 3 mice); (**J**) 2° antibody-treated samples showed background staining.

Both M1-like microglia/macrophages as well as the NK cells are known to eliminate cancer stem cells [37,47]. Therefore, we performed flow cytometry analysis of CD133(+) GBM stem cells in the dissociated tumor cells (UL, red ellipse). We observed that CLp was capable of suppressing the CD133(+) IF by 92% (Figure 7A,B,E,F), whereas TrLp suppressed it by 99% (Figure 7A,C,E,F) [9,43].

As demonstrated before, GBM cells express high levels of CD68 (CD68^high^), whereas the Iba1(+) TAM display much lower CD68 expression (CD68^low^) [14,22]. Therefore, we analyzed the possibility of activation of caspase3 (apoptosis) in the CD68^high^ GBM cells in the tumors after treatment with TrLp or CLp, which could be a result of the observed M1 polarization of TAM and recruitment of activated NK cells, both of which are highly tumoricidal [14,15,48,50]. CLp treatment elicited a 47% increase in active-caspase3 IF in the CD68^high^ GBM cells (UR, within the red rhombus) (Figure 7G,H,K,L). In comparison, TrLp triggered a 96% increase in active-caspase3 IF (Figure 7G,I,K,L). Parallel to the increase in CD68^high^, active-caspase3(+) cells, there was a decrease CD68^high^, active-caspase3(−) cells (blue circle in the LR) (non-apoptotic GBM cells) (Figure 7G–I). In contrast, the Cd68^low^ TAM were active-caspase3(−) and did not change much after treatment with both CLp and TrLp (shown by an arrow in the LR quadrant of each scatter plot) (Figure 7G–I) [14,22]. M1-like microglia/macrophage-derived nitric oxide (NO, which is the product of iNOS activation) and activated-NK cells can both cause induction of p53 in tumor cells, which could lead to this observed caspase3-mediated apoptosis in the CD68^high^ GBM tumor cells to cause tumor shrinkage [52,53,54].

### 2.10. Short-Term Treatment with TrLp and CLp Causes Tumor Shrinkage, but Long-Term Treatment Causes Rescue of Mice

On day 16, after GL261 implantation on day 1 (i.e., the fifth day of the treatment), all the mice from the three short-term treated groups were anesthetized, and an adduct of CD68 Ab linked to a near-infrared (Near-IR) dye Dylight800 (CD68Ab-Dylight800) was intranasally (IN) delivered [14]. After 24 h (i.e., on day 17), all the mice were anesthetized, sacrificed and the brains were extricated. Post-mortem near-IR scanning of those brains revealed that all the mice from the three short-term treated groups harbored smaller tumors (Figure S6A–C), which were on average 61% less in the CLp-treated and 75% less in the TrLp-treated mice than in the Vehicle-treated GBM mice [22]. However, the average tumor load in the TrLp mice was not significantly different from that in the CLp-treated mice (Figure S6A–D).

In our previous studies using GBM-targeted C we were successful in rescuing 50–60% GL261-implanted GBM mice using antibody-targeted C or Curcumin Phytosome Meriva [14]. Similarly, in order to assess the comparative efficacy of CLp and TrLp treatment in longevity and the subsequent survival of GBM mice, we performed a pilot long-term experiment. Similar to the short-term treatments, GBM mice were divided into three groups (*n* = 4 per group) and treated with Vehicle (PBS), CLp (5 mM), or TrLp (1.28 mM+) every 72 h for two months. The prolonged CLp treatment evoked GBM remission in 25% of the GL261-implanted GBM mice, whereas TrLp treatment caused the rescue of 50% of the GBM mice, suggesting higher potency of TrLp than CLp in eliminating GBM tumors in vivo (Figure S6E). Interestingly around day 27, only 25% of the Vehicle-treated GBM mice were alive, in comparison with 50% of the CLp and 100% of the TrLp mice (Figure S6E). Thus TrLp, compared to CLp also significantly increased the longevity of the GBM mice. Bright-field images of the extricated brains of Vehicle-treated morbid mice revealed the presence of large tumors around the implantation site (Figure S6F). In contrast, extricated brains of CLp and TrLp-treated and rescued mice beyond 150 days of survival showed no apparent presence of tumor (Figure S6G,H). This corroborates our short-term tumor-load data (Figure S6A–D), showing that prolonged treatment of TrLp could rescue GBM mice by eliminating GL261-evoked GBM cells and GBM stem cells (Figure 7). Immunohistochemical staining GBM brains revealed an abundance of closely apposed and clustered CD68^high^ GBM cells in the tumor-core area of the Vehicle-treated mice (Figure S7A, yellow ellipse). In sharp contrast, the scar tissue area from the CLp (Figure S7B, red ellipse) and TrLp-treated (Figure S7C, red ellipse) and rescued mice showed the absence of CD68^high^ GBM cells, confirming complete GBM elimination [14,22,32]. These results were also consistent with our previous reports, which showed a reduction in tumor size due to the effects of Tricurin on HPV+ murine and human-derived xenograft tumors in mice [26,27].

## 3. Discussion

Most cancer treatment strategies rely on the understanding that chemotherapeutic agents directly kill cancer cells and this is the only mechanism of cancer elimination. This firm notion makes researchers strive to achieve supra-IC_50_ plasma concentrations of any anticancer drug. Whereas the working concentrations of the chemotherapeutics often elicit side effects, such as elimination of healthy cells and suppression of the immune system, sub-IC_50_ concentrations of any anti-cancer agent are most often believed to be ineffective for treatment [4,10]. Our pharmacokinetic analysis of TrLp-derived C in the plasma revealed a transient peak at 0.61 μM 10 min after TrLp administration, and, thereafter, a more steady concentration of 0.21 μM up to 60 min in the plasma (Figure S3). Though these values are significantly higher than the previously reported plasma concentration of C released from Curcumin Phytosome Meriva^®^ [36], they are still lower than the IC_50_ of TriCurin-associated C for the GL261 cells (5 μM) (Table S1). Quite strikingly, in our experiments, treatment with TrLp did indeed cause elimination of CD68^high^ GBM cells in vivo, suppression of CD133+ GBM stem cells, and tumor remission in 50% of mice that originally harbored established GBM (Figures S3 and S6, Figure 4 and Figure 7) [14,21,22,28,32]. How is this even possible? Intriguingly, our studies here show that despite such relatively low plasma concentrations of TriCurin-associated C (in the nano-molar range), TrLp is able to (i) cause a major stimulation of the innate immune system, such as repolarization of the TAM, from the tumor-promoting M2-like form to the tumoricidal M1-like state, and also (ii) trigger intra-tumor recruitment of NK cells from the periphery. These activated NK cells play a major role in maintaining the M1-like state of the TAM and also in the killing of tumor cells [51]. Thus, TrLp could indirectly eliminate tumor cells by recruiting the immune cells [13,14,15,16,31,46,55]. Interestingly, TrLp (1.28 mM of C), which contains a lower concentration of C than in CLp, caused a greater level of stimulation of the innate immune system, greater elimination of GBM and GBM stem cells, and tumor remission in 50% of the GBM mice, whereas administration of CLp (containing 5 mM of C) resulted in a lesser degree of innate immune cell stimulation, lower elimination of GBM and GBM stem cells, and remission in 25% of the GBM mice (Figures S3–S6, Figure 5, Figure 6 and Figure 7) [13,14,15]. It is noteworthy that R and E (two other components of TriCurin) have been reported to be effective oncoimmunotherapeutic agents [16,56,57]. Thus, these two components may also have a strong contribution in the observed synergistic relationship between C, R and E in TriCurin and therefore they are likely to make TrLp a more potent immunotherapeutic agent than C.

We have designed and characterized a novel liposomal formulation of TriCurin (TrLp) and performed comparative studies with liposomal curcumin (CLp) to first test for the direct anti-tumor efficacy of TrLp in cultured mouse GL261 GBM cells before embarking on the in vivo experiments discussed above (Figure 1 and Figure S1, Table S1) [14,20,21,22,26,27,58]. These experiments also enabled us to delineate the involvement of the cell cycle inhibiting transcription factor p53 in direct killing of GL261 cells without the involvement of the immune cells. We observed that both CLp (10 μM) and TrLp (10 μM+), with the same concentration of C (in the IC_50_ range), triggered induction of both p53 and acetyl-p53 (activated p53), and activation of caspase3, but the TrLp-evoked changes were significantly greater than those caused by CLp (185%, 406% and 1895% greater induction of acetyl-p53, p53 and active caspase3, respectively) (Figure 2 and Figure 3) [49]. This likely involvement of p53 for TrLp-mediated elimination of GL261 cells in vitro could be the result of our previously demonstrated C-mediated inhibition of the oncoprotein NF-κB that causes inhibition of p300-HAT [22,26,27,41]. Thus C-evoked inhibition of NF-κB would trigger p300-HAT-mediated acetylation and stabilization of p53, thereby causing downstream activation of caspase3 and elimination of cultured GL261 cells (Figure 2 and Figure 3, Figure S1, Table S1) [41,42]. Additionally, the TrLp-induced p53 activation can cause suppression of CD133(+) and SOX2(+) GBM stem cells (in GL261 culture), which are known to cause tumor recurrence even after the available therapies (Figure 4) [9,24,44,45]. However, unless a transient and pre-detection bolus of C in the IC_50_ range occurs in vivo, the entire mechanism of elimination of GBM and GBM stem cells may not involve direct action of C on signaling proteins such as p53. Rather, as discussed earlier, an involvement of the innate immune system might drive GBM elimination.

The reason why TrLp is remarkably more effective than CLp is not fully clear but previous studies and our data indicate that C (in combination with R or E or in TriCurin) is more potent and is possibly stabilized by R and/or E, which allows a higher intracellular concentration of C to be available for exerting its anti-tumor effects (Figure S2) [26,59,60,61,62]. Our pharmacokinetic data established greater stability and bioavailability of C when present as TrLp by showing that the plasma concentration of C after i.p injection of TrLp was several times greater than previously reported values for lipid attached-C (Curcumin Phytosome Meriva^®^) in rat (Figure S3) [36]. However, the chemical nature of the protective interactions of R and E on C (in TriCurin) is still unknown and it warrants spectroscopic studies [59,60].

Established GBM is highly metastatic and is particularly difficult to treat because of tumor heterogeneity, limited ability of therapeutic agents to cross the blood-brain barrier, and acquired resistance of cancer cells to standard therapies [63,64]. Given the serious side-effects of current standard of care for GBM and the fact that brain tumor incidence and mortality have significantly increased during the past decades, there is an urgent need to combat this aggressive disease using alternative therapeutic agents such as TriCurin [4,5,6,7,8,9,10,11,26,27,65]. Recently, immunotherapy has come into prominence, but, though promising, it suffers from some serious side effects [12,49,66]. Our earlier studies have demonstrated that TriCurin is harmless to normal tissue and selectively toxic against tumors [26,28]. Previous reports have demonstrated the prophylactic effects of C on tumor incidence and formation [28,59]. However, most GBM patients are diagnosed and treated only when the tumors are already established and the treatment regimens consisting of surgical debulking of tumor followed by chemo and radiation therapies are rarely successful in rescuing patients [2,3,4,11]. In contrast, here we demonstrate TrLp’s superior capacity in eliminating established GBM tumors, possibly by stimulating the innate immune cells [14,21,22,28,32,65]. This report, taken together with our previous studies, demonstrate that TrLp is potent, relatively stable, and safe [26,27]. Thus, TrLp can serve as a promising onco-immunotherapeutic agent that may eliminate established GBM tumors in patients, who currently surrender to the available palliative treatments, which only extend their lives by a few months [1,2,3,64].

## 4. Methods and Materials

### 4.1. Animals

Adult C57BL/6 male mice (2–4 months old) were used for the experiments. Animals were bred in the College of Staten Island (CSI) Animal Care Facility and maintained on a 12-h light/dark cycle with *ad libitum* access to food and water. All procedures performed in studies involving animals were in accordance with the ethical standards of the Institutional Animal Care Committees (IACUC) at the College of Staten Island (approval # 11-008).

### 4.2. Curcumin & TriCurin

Curcumin (C) (≥98% curcuminoid content) (CAS number 458-37-7) (ThermoFisher Scientific, Springfield Township, NJ, USA); stored at room temperature under nitrogen); solution was prepared as described earlier [26,28]. TriCurin solution was prepared from curcumin, (−)-epicatechin gallate (E), and resveratrol (R) (Thermo Fisher Scientific, Springfield Township, NJ, USA) at the molar proportion C:E:R: 4:1:12.5 as reported earlier [26].

### 4.3. Preparation of TriCurin Lipososme (TrLp) and and Curcumin Liposome (CLp)

Curcumin (C), epicatechin-gallate (E), and resveratrol (R) were dissolved in DMSO (for C and R) and deionized water (for E) to yield stock concentrations of 40 mM (C), 50 mM (E) and 200 mM (R). Aliquots of the stock solutions corresponding the required masses of C, E and R were added in increments to sterile PBS under vigorous vortexing to yield final concentrations of 1.28 mM (C), 320 μM (E), and 4 mM (R) in PBS containing 5% DMSO (termed as 1.28 mM+ TriCurin). Soy phospholipid mixture (# 541601G, Avanti Polar Lipids, Inc., Alabaster, AL, USA) corresponding to four-times the mass of TriCurin (C + E + R) was added to the TriCurin solution. The mixture was protected from light and vigorously sonicated at medium setting for 10 min at −20 °C three times [58], holding it on ice for 5 min between sonication cycles. The uniform emulsion (TrLp) generated was then divided into aliquots, and each aliquot was purged thoroughly with nitrogen and stored, protected from light at −20 °C. CLp was prepared in the same way using a 5 mM curcumin solution and soy phospholipid mixture (corresponding to four-times the mass of C) in PBS, processed, and stored at −20 °C.

### 4.4. Purification of Liposomes and Analysis of Liposomal Dimensions by Dynamic Light Scattering (DLS)

One milliliter of a liposomal suspension (CLp & and TrLp) was centrifuged at 10,000× *g* for 10 min to remove any insoluble material. The supernatant was loaded onto a Hiprep 26/10 desalting column (GE Life Sciences, Marlborough, MA, USA). The liposomes were eluted using phosphate buffered saline (PBS, pH 7.4) at 2 mL/min while monitoring the absorbance at 430 nm to detect curcumin. All curcumin-containing fractions were pooled. Each purified liposome suspension was filtered through a 0.45-μ filter before being analyzed by Dynamic light scattering (DLS). DLS was performed using a standard laser light scattering spectrometer (BI-200SM) equipped with a BI-9000 AT digital time correlator (Brookhaven Instruments Co, Holtsville, NY, USA) to measure the hydrodynamic diameter distributions.

### 4.5. Confocal Imaging of Liposomes

After preparing CLp (5 mM) and TrLp (1.28 mM+) drops of these liposomes were placed on glass slides and protected with cover slips. Confocal Imaging for the verification of curcumin encapsulation was conducted at 540 nm (curcumin’s fluorescence emission maximum) using a SP2 confocal microscope (Leica Microsystems, Inc., Buffalo Grove, IL, USA) from multiple randomly chosen fields.

### 4.6. Cell Culture

GL261 mouse glioblastoma cells were cultured according to our earlier report [14,22].

### 4.7. Determination of IC_50_ Using WST-1 Assay

Stock solutions of curcumin, TriCurin, CLp and TrLp were prepared as mentioned earlier. Each stock was serially diluted in RPMI plus 1x insulin-transferrin-selenium (ITS) supplement (ThermoFisher Scientific, Springfield Township, NJ, USA) and added to GL261 cells cultured in a 96-well plate to determine dose-dependent cell viability and IC50 values using the WST-1 assay [14,26].

### 4.8. Clonogenic Assay

GL261 cells were plated in 6-well plates and treated with CLp (10 µM) and TrLp (10 µM+) for 96 h. After 96 h, the cells were trypsinized and single cell suspensions were made as described earlier [14]. The number of viable cells in these suspensions were counted using trypan blue dye exclusion test and 50 viable cells (trypan blue negative) from each treatment were plated in 6-well plates and allowed to grow in complete RPMI medium for 14 days. The cells were washed with PBS, fixed in 6% glutaraldehyde, and then stained with 0.5% crystal violet in 6% glutaraldehyde (*w*/*v*). The cells were then rinsed with tap water and allowed to air dry. Images of each well were acquired and the crystal violet-stained colonies were counted using Image J (National Institutes of Health, Bethesda, MD, USA).

### 4.9. Staining of GL261 Cells and GBM Tissue for Flow Cytometry

For the purpose of signaling studies, GL261 cells were treated with CLp (10 µM) and TrLp (10 µM+) in RPMI plus 1x ITS supplement for 6 h. Cells were washed in PBS (2×) and single-cell suspensions were prepared for immunostaining as reported earlier [14]. GBM tissues obtained after five days of intraperitoneal CLp (5 mM) and TrLp (1.28 mM+) treatment of GBM-implanted mice were also processed for flow cytometry as detailed previously [14]. The fixed cells were counted and stored in 2% formaldehyde at 4 °C. Around 2 million fixed cells from each animal were used for immunostaining as described earlier [14]. After each antibody incubation, the samples were pelleted and resuspended in wash buffers. Antibodies against p53 (rabbit IgG) (SC-6243) (1:100), Acetyl p53 (rabbit IgG) (GTX88013) (1:100), CD68 (rabbit IgG) (SC-9139) (1:100), Active-Caspase3 (rabbit IgG) (Asp 175) (9661S) (1:100), CD133 (goat IgG) (SC-19365) (1:50), SOX2 (goat IgG) (SC365823) (1:50), Iba1 (goat IgG) (C20) (sc28530) (1:50), iNOS (rabbit IgG) (NOS2 sc-651) (1:100), Arginase1 (ARG1) (rabbit IgG) (sc-20150) (1:100), and NKp46 (rabbit IgG) (sc-292796) (1:75) were used for staining. Cells were then treated with the following secondary antibodies: Alexa Fluor goat anti rabbit 488, Alexa Fluor goat anti rabbit 568, Alexa Fluor rabbit anti goat 568 and Alexa Fluor goat anti mouse 568 (Invitrogen). Flow cytometry was performed using an Accuri C6 flow cytometer (BD) as reported earlier [14]. The p53/acetyl-p53, CD68/Active Caspase3, iNOS/Iba1, and ARG1/Iba1 double-stained fluorescent events from the cells appearing as sub-populations in the upper right (UR) quadrant within the coordinates 520 nm (green for p53, Active-Caspase3, iNOS, and ARG1) (FL1-A) and/or 580 nm (red for Acetyl-p53, CD68, and Iba1) (FL2-A) were considered. CD133, SOX2, and NKp46 single-stained events appearing in the upper left (UL) quadrant within the 580 nm (red) coordinate were used for quantification. Integrated fluorescence (IF) intensity was measured for comparison between groups by multiplying the number of positive events (single stained or double stained cells) by the mean fluorescence intensity.

### 4.10. Immunocytochemical Staining of SOX2(+) GL261 Cells for Studying Curcumin Internalization and/or Stabilization

Gl261 cells were plated and processed for immunocytochemistry as reported earlier [14,26]. Treatment of GL261 cells with CLp and TrLp and 6-hour incubation (protected from light) was conducted as mentioned in Section 4.9. Cells attached to cover-slips in the wells were washed with 10 mM phosphate-buffered saline (PBS), fixed in 4% (*w*/*v*) paraformaldehyde, washed three times with PBS, blocked in 10% goat serum in PBS plus 0.1% Triton X-100, and then immunostained using anti-SOX2 Ab (mouse IgG) (sc-365823) (1:50), followed by a secondary Ab (Alexa Fluor 568 goat anti-mouse, 1:1000, Invitrogen) [14,26]. The cells were subsequently treated with HOECHST33342 (10 µg/mL) for 30 min at room temperature, mounted, and imaged using confocal microscopy with filters set at 568 nm (excitation) and 580 nm (emission), respectively for SOX2. Curcumin fluorescence (green) was imaged using a Leica SP2 confocal microscope with the filters set at 488 nm (excitation) and 540 nm (emission), respectively. The data were then analyzed and quantified using ImageJ.

### 4.11. Extraction of Curcumin from the Plasma of Mice Treated with TriCurin Liposome and Quantification by HPLC Analysis

Tumor-naive mice treated (i.p.) with TrLp (200 μL, 1.28 mM+) were sacrificed and blood samples from the heart were collected in heparinized tubes 0 min, 10 min, 20 min, 30 min, and 60 min (*n* = 3 animals at each time point) after injection. The tubes were centrifuged to pellet the cells, and the supernatants (plasma) were transferred into labeled tubes and kept on ice. For long-term storage, the samples were flash-frozen in an ethanol-dry ice bath and stored at −20 °C. The plasma from each mouse was extracted with 0.5 mL of acetonitrile:water mixture (70:30) with vigorous mixing, centrifuged, and the supernatant was transferred to a fresh Falcon tube. Acetonitrile:water mixture (70:30) (0.5 mL) was added to the pellet and vigorously mixed. After centrifugation, the new supernatant was pooled together with the first supernatant and evaporated at 45 °C under a stream of nitrogen. The residue was dissolved in 50 μL of Acetonitrile: water mixture (70:30) and 20 μL of this solution injected into the HPLC analyzer fitted with a C18 reverse-phase column. The column was eluted using a 30–70% gradient of acetonitrile in water and 0.1% trifluoroacetic acid (TFA). Curcuminoids were eluted as a triplet at a retention time of ~13 min [28].

### 4.12. Implantation of GL261 Cells in Mice

GL261 mouse glioblastoma cells (10^5^) were intracranially implanted in mice according to our earlier report [14].

### 4.13. Preparation of Dylight 800-CD68 Ab Adduct and Intranasal Treatment of GBM Mice

The Dylight 800-CD68 Ab adduct was synthesized according to our earlier report [14]. Intranasal treatment of the GL261 implanted GBM mice was performed also according to our previous report [14].

### 4.14. Treatment of Animals

C57BL/6 male mice were implanted with 10^5^ GL261 cells (*n* = 21) [14,22]. These were randomly divided into three groups: TrLp (1.28 mM+), CLp (5 mM), and PBS (Vehicle) (*n* = 7 for each group). Each of these groups was sub-divided into “short term treatment” (*n* = 3) and “long term treatment” (*n* = 4) cohorts. “Short term (5-day) treatment” for all three groups began on day 12 after the implantation of GL261 cells on day 1, and the mice were treated every 24 h for five days with intraperitoneal (i.p.) CLp, TrLp or PBS (200 μL of each, per mouse). For the short-term-treated mice, the intranasal Dylight-CD68 Ab delivery was performed on the fifth day (i.e., day 16) as detailed earlier [14], and mice from all the three groups were sacrificed on the sixth day (i.e., on day 17) and their brains were scanned to determine the tumor loads as previously described using the ImageJ software [14,22].

Long-term treatment for all three groups also began 12 days after the implantation of GL261 cells on day 1, and the mice were treated every 72 h for 60 days also via i.p. administration of the respective agents. The rescued mice from GBM were allowed to live till day 150 and then sacrificed for the analysis of the scar tissue [14].

### 4.15. Immunohistochemistry

The post-mortem processing, sectioning and immunohistochemical staining with iNOS, ARG1, Iba1, and CD68 and subsequent analysis of the extricated brains were performed as detailed in our previous reports [14,22,26,27]. For NKp46 immunostaining of the short-term treated GBM brains, anti-NKp46 antibody (rabbit IgG) (sc-292796) (1:50) was used followed by secondary Ab (Alexa Fluor 568 goat anti-rabbit, 1:1000, Invitrogen). Confocal imaging was conducted using a Leica SP2 microscope from multiple randomly chosen fields and the ImageJ software was used to quantify the fluorescence intensities as described earlier [14,22].

### 4.16. Statistical Analysis

Two-tailed t-tests with unequal variance were used while comparing between two groups and one-way ANOVA with Tukey for post-hoc analysis was employed to compare among three groups (*p* ≤ 0.05 was considered as significant).

## Supplementary Materials

The following are available online.
