# Supplementary material for: Liposomal TriCurin, A Synergistic Combination of Curcumin, Epicatechin Gallate and Resveratrol, Repolarizes Tumor-Associated Microglia/Macrophages, and Eliminates Glioblastoma (GBM) and GBM Stem Cells
Source: Molecules. 2018 Jan 18;23(1):201. doi: 10.3390/molecules23010201 (PMC6017476; doi:10.3390/molecules23010201)
Supplement: Supplementary file 1 [file molecules-23-00201-s001.pdf]

SUPPLEMENTARY INFORMATION

Liposomal TriCurin, A Synergistic Combination of Curcumin, Epicatechin Gallate and Resveratrol, Repolarizes Tumor-Associated Microglia/Macrophages, and Eliminates Glioblastoma (GBM) and GBM Stem Cells

Sumit Mukherjee*[§], Juliet Baidoo*, Samay Sampat[¶], Andrew Mancuso*[§], Lovena David[§], Leah S. Cohen[§], Shuiqin Zhou[§], and Probal Banerjee[§fl]

GL261 Cells	Curcumin (C)	TriCurin	CLp	TrLp
**IC50 (μM)**	15	4+	16	5+

Table S1. TrLp is more potent than CLp in eliminating GL261 cells.

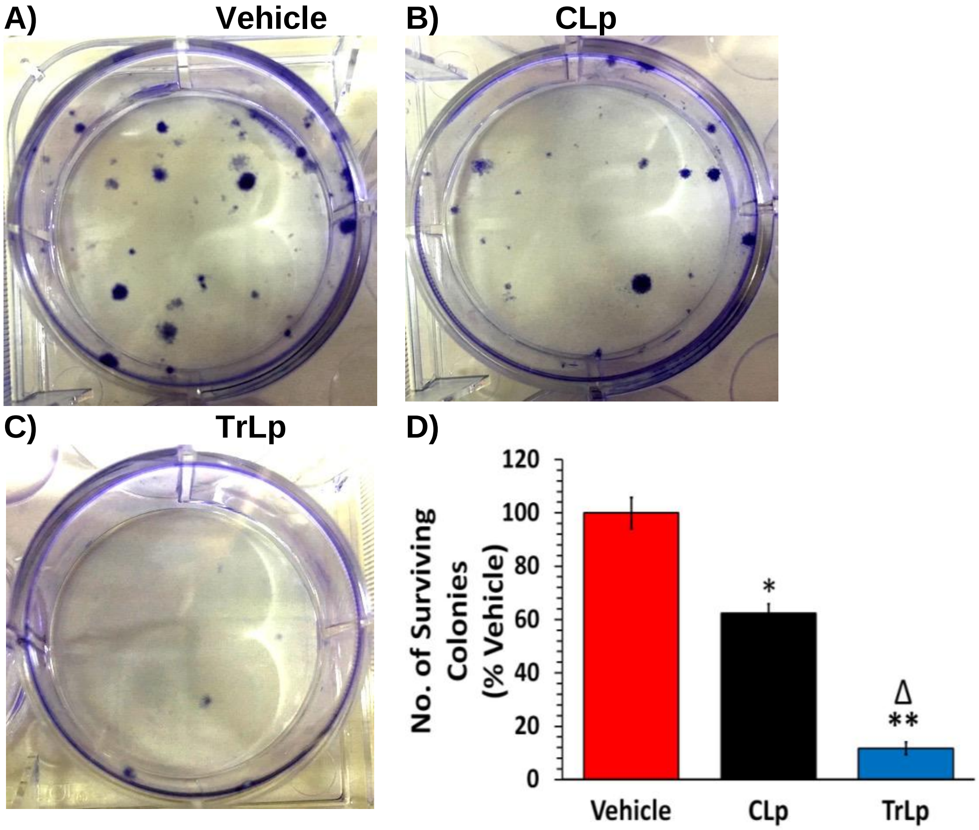

Figure S1. TrLp is more potent than CLp in attenuating the clonogenic potential of GL261 cells. The effects of Vehicle (PBS), CLp (10 μM), and TrLp (10 μM+) on the clone-generating capacity of 6 h-treated and surviving GL261 cells were assessed after 15 days. **(A)** Discrete surviving GL261 colonies were visible in the Vehicle-treated group. **(B)** In contrast, the surviving CLp-treated GL261 colonies were sparse. **(C)** In the TrLp-treated group, almost no surviving GL261 colonies were observed. **(D)** Compared to the vehicle-treated group, the number of GL261 colonies was reduced by 38% with CLp (*p = 0.03) and by 88% with TrLp (**$p = 5.12x10^{-3}$). The clonogenic potential in the TrLp-treated cells was 51% lower than that in the CLp-treated group (Δ $p = 6.96x10^{-3}$). (**B, C)** The graph represents data (mean ± S.E.M.) obtained from Vehicle (n = 3), CLp (n=3) and TrLp (n=3).

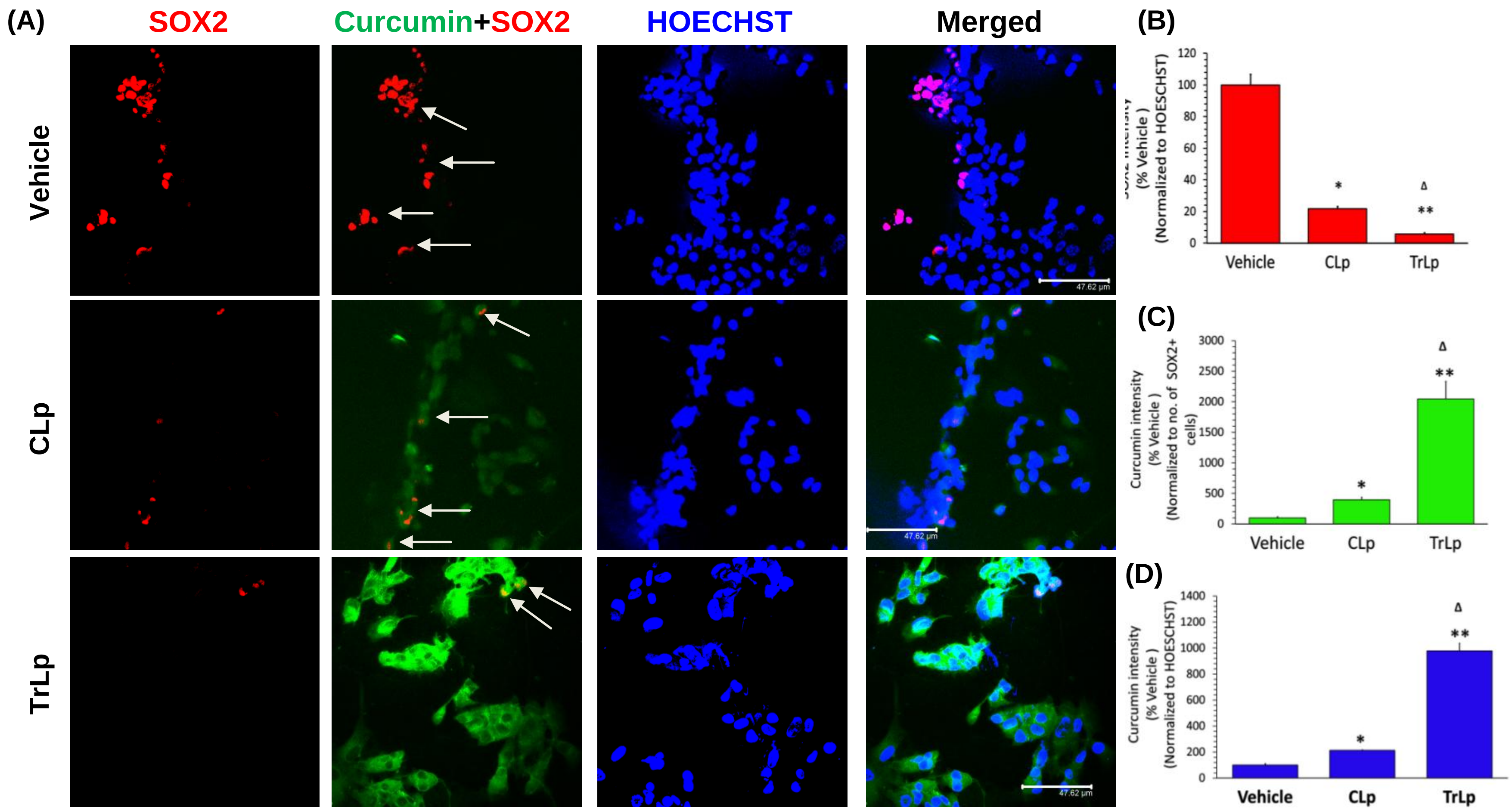

Figure S2. TrLp causes greater accumulation of curcumin in GL261 and GL261 stem cells in culture. GL261 Cells were incubated for 6 hours with Vehicle (PBS), CLp (10 µM) and TrLp (10 µM+). Following incubation, immunocytochemistry with anti-SOX2 antibody was performed to assess the cellular uptake of curcumin (C) into the SOX2(+) GL261 stem cells. **(A)** Cells of the Vehicle group displayed strongly stained sparse populations of SOX2(+) GL261 stem cells (red) but no detectable green staining (due to absence of internalized C, white arrows). (**A**, top row). Cells incubated with CLp displayed a 78% decrease in SOX2 fluorescence (*p= 4.8 x 10^{-5}, CLp versus Vehicle) (**A**, top and middle rows; **B**), while showing a 296% increase in C's fluorescence normalized to the number of SOX2(+) cells (white arrows) (*p= 2.3 x 10^{-3}, CLp versus Vehicle) (**A**, top and middle row ; **C**). TrLp-treatment induced a 94% suppression in SOX2 fluorescence (**p=1.7x10^{-4}, TrLp versus Vehicle; ∆ p=3.4x10^{-4}, TrLp versus CLp) (**A** and **B**), while causing a 1947% increase in C's fluorescence normalized to the number of SOX2(+) cells (white arrows) (**p=2.5x10^{-3}, TrLp versus Vehicle; ∆ p = 1.1x10^{-3}, TrLp versus CLp) (**A** and **C**). **(D)** Curcumin uptake in the GL261 cells in general also increased by 114% for CLP and by 878% for TrLp (**p=1.3x10^{-4}, TrLp versus Vehicle; ∆ p = 2.1x10^{-5}, TrLp versus CLp; *p =7.3 x 10^{-5}, ClP versus Vehicle). This indicates that TrLp may augment uptake of C into both SOX2(+) GL261 stem cells (white arrows) and also the GL261 cells . The fluorescence of C was imaged at its emission maximum at 540 nm. Data (mean ± S.E.M.) obtained from Vehicle (n=4), CLp (n=4) and TrLp (n=4) treatment. (Scale bar: 47.62 µm).

(A)

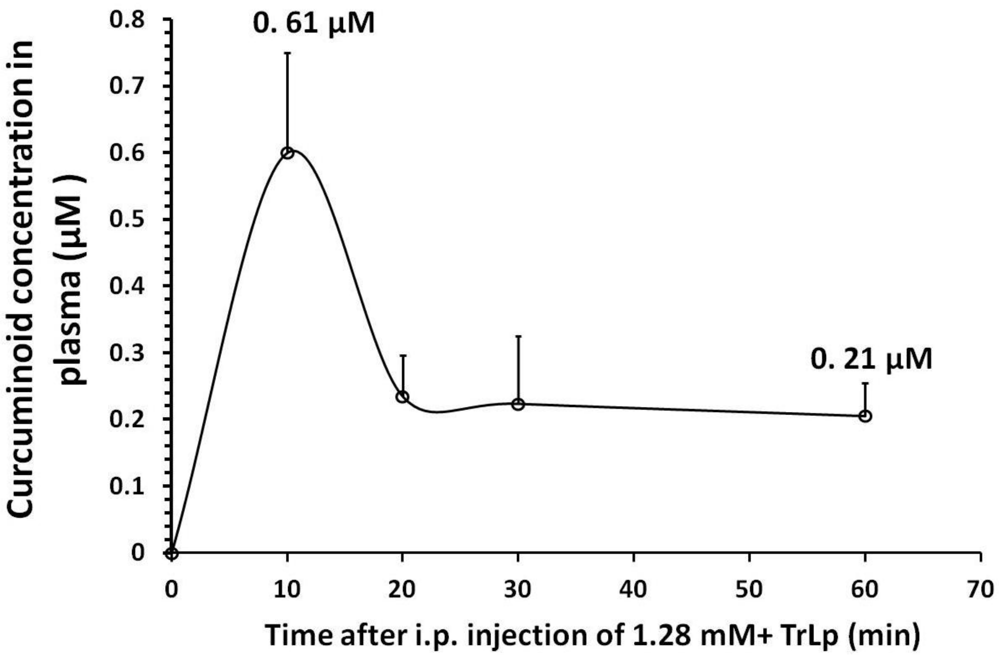

Figure S3. Pharmacokinetic assessment of curcuminoids (C) in the plasma of TrLp-injected mice. Pharmacokinetic analysis of TrLp via HPLC revealed curcuminoids (C) as a closely spaced triplet with the strongest peak eluting at ~13 min [1]. 10 min after the i.p injection of 200 µL of 1.28 mM+ TrLp (containing 0.256 micromoles of C), the C concentration peaked in plasma (0.61 µM) and remained nearly constant at ~0.2 µM from 20 to 60 minutes after injection.

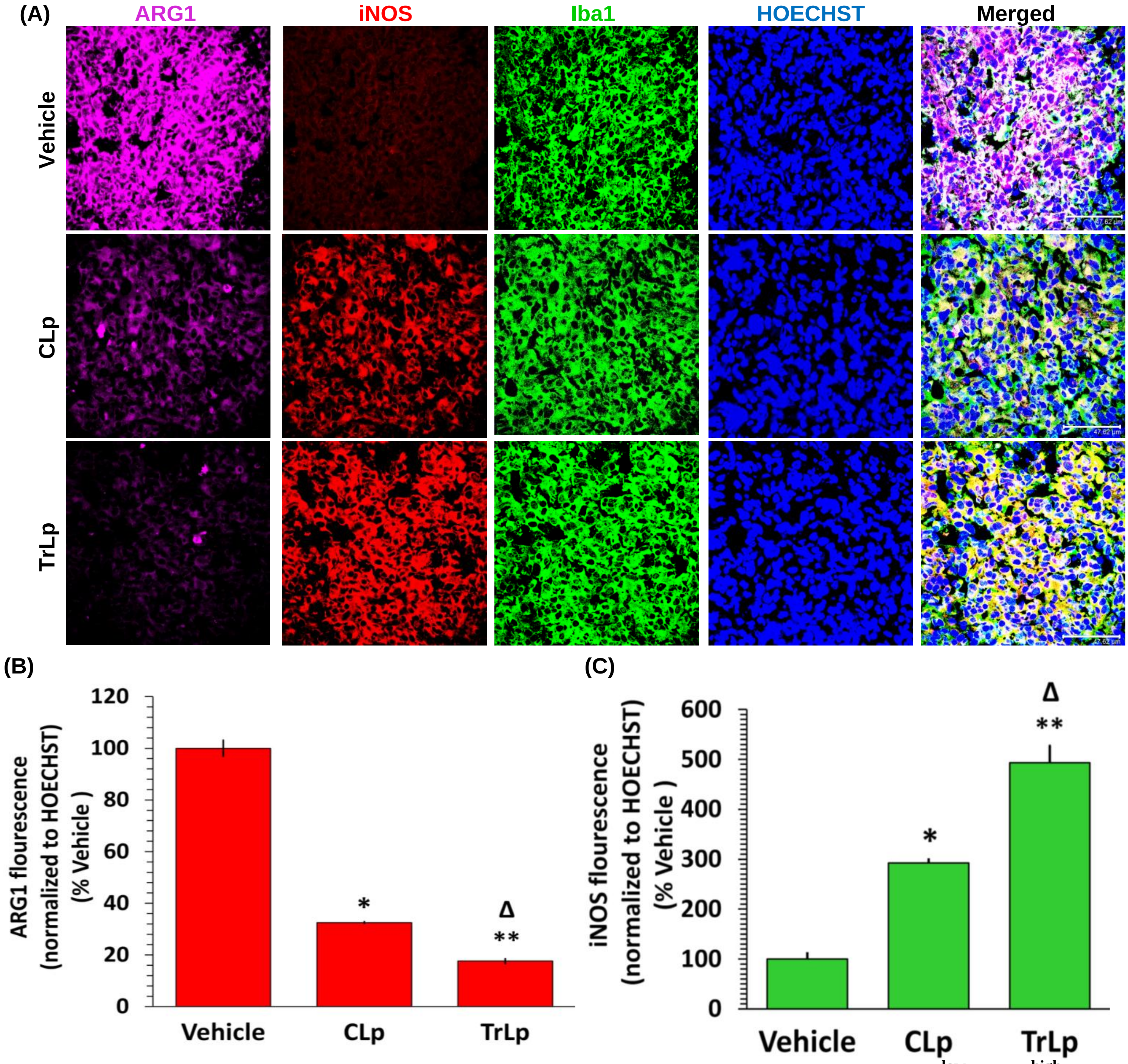

Figure S4. TrLp is significantly more potent than CLp in causing repolarization of iNOS^{low}, Arg1^{high} M2-like TAM to iNOS^{high}, Arg1^{low} M1-like TAM. Corroborating the flow cytometry data presented in **Figure 5**, brain sections harboring the GBM tumor from the three groups of mice were triple-stained with Iba1 (green), iNOS (red) and ARG1 (magenta) antibodies. **(A)** The Iba1+ TAM in the Vehicle-treated GBM sections displayed weak iNOS staining but strong ARG1 staining (**A**, top row). The CLp-treated mice exhibited a 67% decrease in ARG1 (*p= 6.5 x 10^{-7}, CLp versus Vehicle). In contrast, the TrLp-evoked suppression of ARG1 in the TAM was 82% in the TrLp sections (**p=4x10^{-6}, TrLp versus Vehicle; Δp=2.9x10^{-7}, TrLp versus CLp) (**A** and **B**). In contrast, the Iba1+ TAM in the CLp-treated mice showed a 192% increase in iNOS (*p= 6.7x10^{-6}, CLp versus vehicle) (**A**, top row). Moreover, the TrLp-evoked increase in iNOS was 393% in the TrLp group (**p=3.5x10^{-5}, TrLp versus Vehicle; Δp=2.4x10^{-3}, TrLp versus CLp) (**A** and **C**). The graphs represent data (mean ± S.E.M.) obtained from Vehicle (n=3), CLp (n=3) and TrLp (n=3). Three random sections per mouse were used for imaging (Scale bar: 47.62 µm).

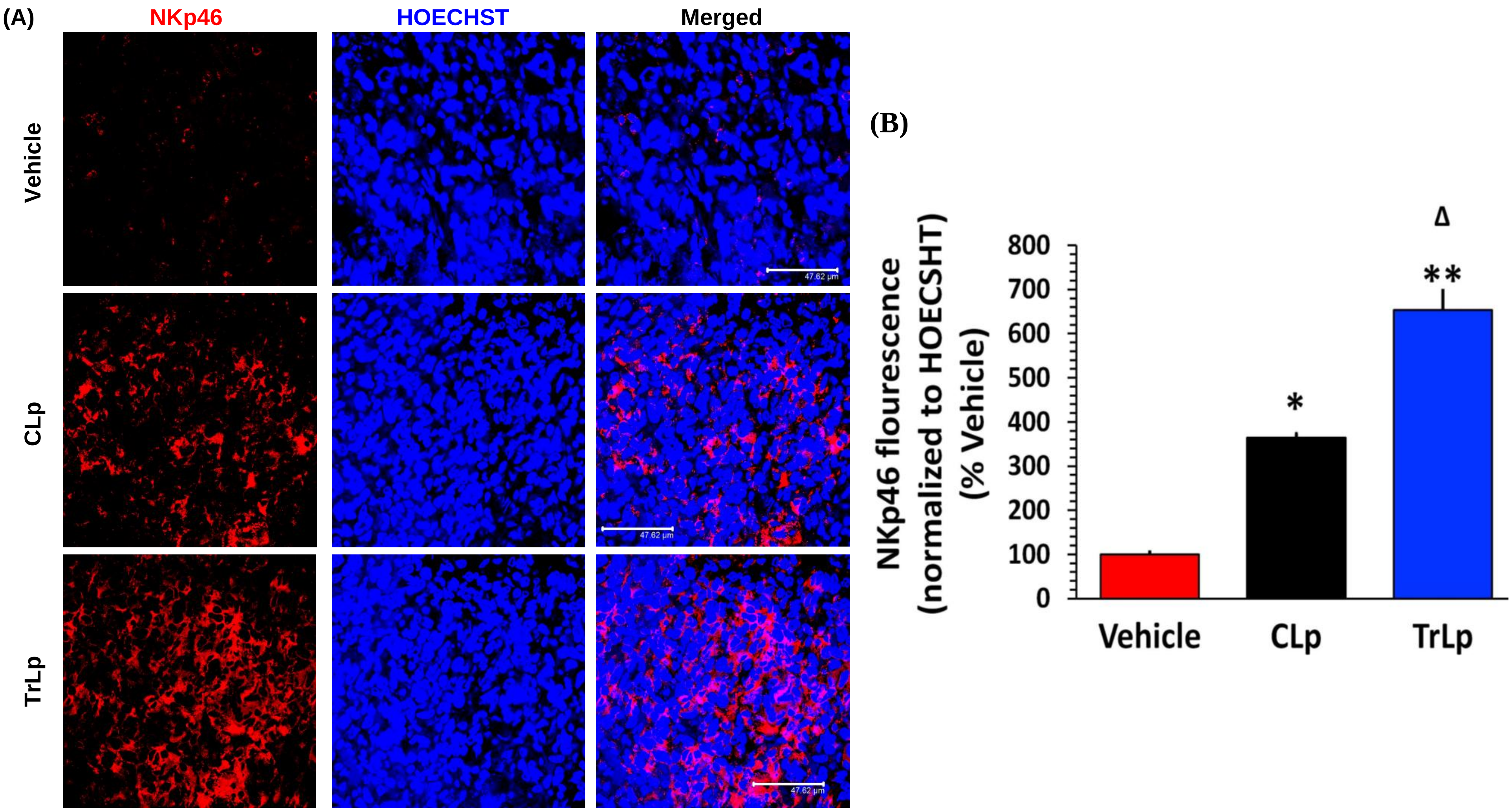

Figure S5. TrLp is significantly more potent than CLp in causing intra-tumor recruitment of NKp46(+) NK cells. Corroborating the flow cytometry data presented in **Figure 6**, multiple, randomly chosen brain sections harboring the GBM tumor from the three groups of mice were immunostained with NKp46 antibody (red). **(A** and **B)** The GBM sections from the vehicle-treated mice exhibited weakly stained and sparse NKp46+ NK cells (**A** top row and **B**). The GBM sections from the CLp-treated mice showed a 264% increase in NKp46 fluorescence (*p= 1.8×10^{-7}, CLp versus Vehicle) **(A** middle row and **B**). In comparison, the NK46 staining of the TrLp-treated mice showed a 553% increase in NKp46 fluorescence (**p=1.6×10^{-5}, TrLp versus Vehicle; Δp=6.4×10^{-4}, TrLp versus CLp) (**A** and **B**). The graphs represents data (mean ± S.E.M.) obtained from Vehicle (n=3), CLp (n=3) and TrLp (n=3). Three random sections per mouse were used for imaging (Scale bar: 47.62 µm).

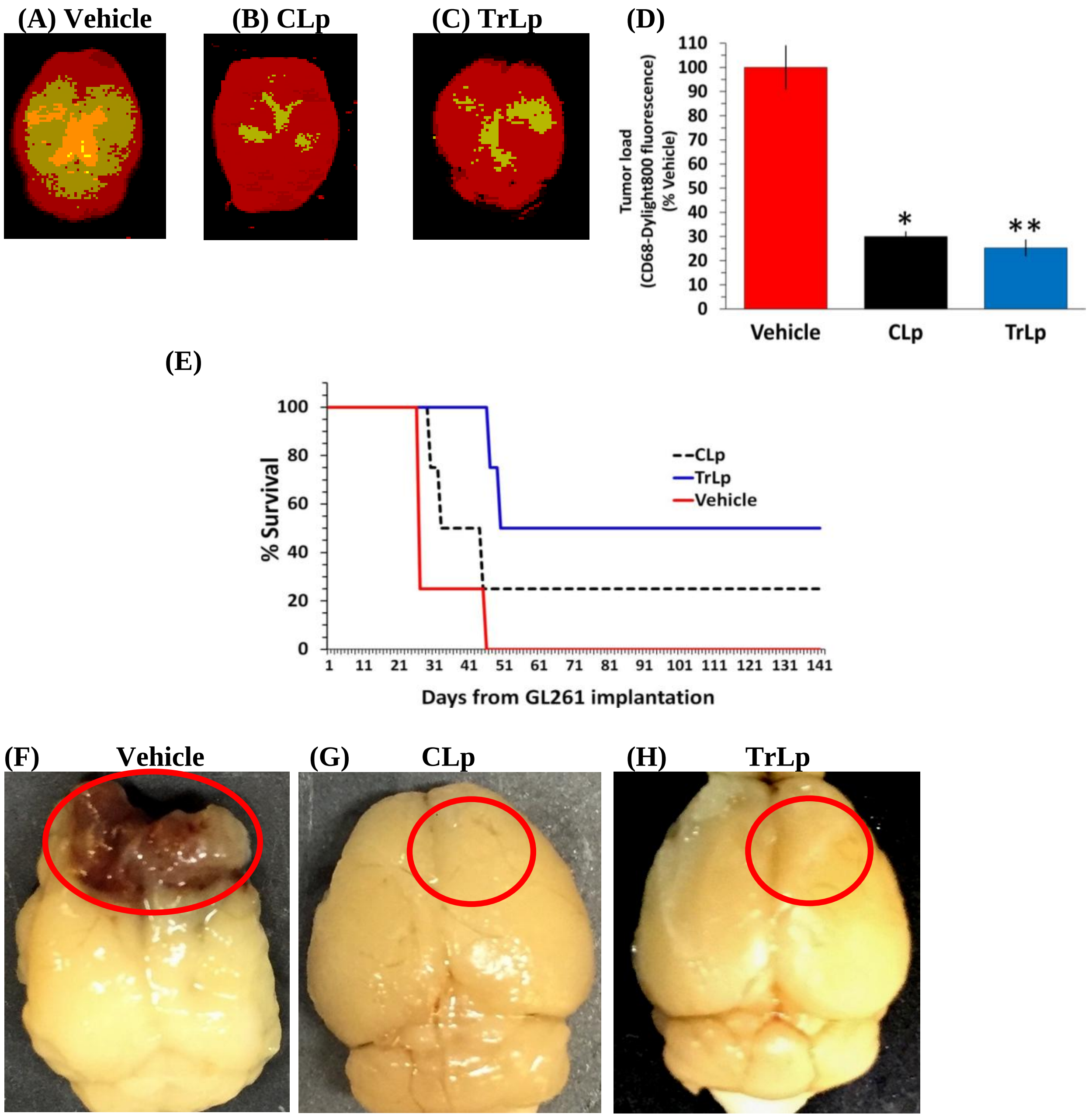

Figure S6. Both CLp and TrLp cause a decrease in tumor load after short-term treatment, but TrLp is more potent in causing tumor remission after long-term treatment. (A-D) The five day-treated (short-term) GBM mice from the three groups (Vehicle, CLp and TrLp) were intranasally (IN) infused with CD68Ab-Dylight conjugate containing 60 µg (400 pmole) of CD68 antibody on day 16 and sacrificed on day 17 [2]. The extricated brains were near-IR scanned to determine the tumor load (800 nm; pseudocolored green) [2,3]. **(A, D)** The tumor regions of the Vehicle-treated mice are bulky, covering large areas of the brains (green). **(A-D)** Compared to the Vehicle-treated group, the tumor load in the CLp-treated GBM mice (green) is 61% less (*p = 1.54×10^{-3}) and that in the TrLp-treated GBM mice is 75% less (**p = 1.40×10^{-3}). The graph represents data (mean ± S.E.M.) obtained from Vehicle (n = 3), CLp (n = 3) and TrLp (n = 3). **(E)** In the survival experiments (long-term treatment), the GBM mice were randomly assigned to three groups, and then treated for 60 days ((i.p. 200 µl, every 72 hours) from day 12 with Vehicle (PBS) or CLp (5 mM) or TrLp (1.28 mM+) (n = 4 for each group). The CLp treatment caused remission in 25% of the GBM mice (*p = 2.34×10^{-7}), whereas the TrLp treatment caused remission in 50% of the GBM mice (**p = 8.93×10^{-27}, TrLp versus Vehicle-treated; Δ p = 6.00×10^{-12}, TrLp versus CLp). **(F-H)** The extricated brains from Vehicle-treated GBM mice (morbid) and the rescued mice from CLp and TrLp treatments (around day 150) were inspected without fixing and brightfield images were acquired. **(F)** Large tumors were visible in the Vehicle-treated GBM mouse brains (red ellipse). **(G, H)** In contrast, the rescued mice from CLp- and TrLp-treated groups did not show any sign of tumor (red ellipse).

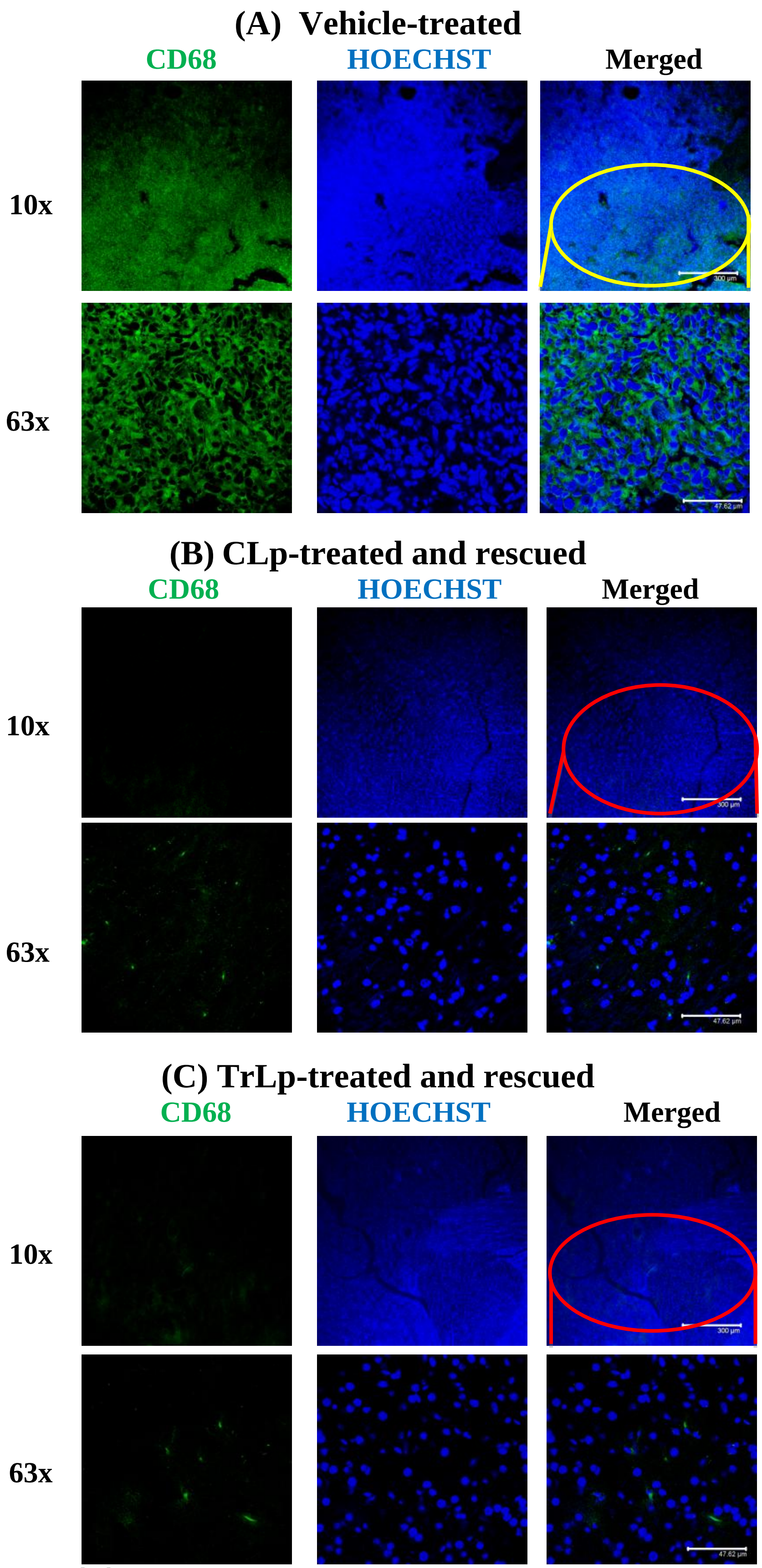

Figure S7. Absence of CD68^{high} tumor cells in the scar tissue area in the rescued mice. **(A)** The GL261-evoked GBM tumor-core (yellow ellipse) had an abundance of CD68^{high} tumor cells. **(B)** The scar tissue area (red ellipse) of the CLp-treated and rescued mouse lacked CD68^{high} GBM tumor cells. **(C)** The scar tissue area (red ellipse) in each of the TrLp-treated and rescued mice was also completely devoid of CD68^{high} GBM tumor cells. (Scale bars: 300 µm (10x) and 47.62 µm (63x)).

References:

1. Purkayastha, S., Berliner, A., Fernando, S.S., Ranasinghe, B., Ray, I., Tariq, H., and Banerjee, P. . Curcumin blocks brain tumor formation. *Brain Research* **2009**, *1266C*, 130-138.
2. Mukherjee, S., Baidoo, J., Fried, A., Atwi, D., Dolai, S., Boockvar, J., Symons, M., Ruggieri, R., Raja, K., and Banerjee, P. Curcumin changes the polarity of tumor-associated microglia and eliminates glioblastoma. *International Journal of Cancer* **2016**, *139*, 2838-2849.
3. Langone, P., Debata, P.R., Inigo, J.D.R., Dolai, S., Mukherjee, S., Halat, P., Mastroianni, K., Curcio, G.M., Castellanos, M.R., Raja, K., and Banerjee, P. Coupling to a glioblastoma-directed antibody potentiates anti-tumor activity of curcumin. *International Journal of Cancer* **2014**, *135*, 710-719.
